# Preparation and Characterization of Thermoresponsive Poly(*N*-isopropylacrylamide-co-acrylic acid)-Grafted Hollow Fe_3_O_4_/SiO_2_ Microspheres with Surface Holes for BSA Release

**DOI:** 10.3390/ma10040411

**Published:** 2017-04-14

**Authors:** Jing Zhao, Ming Zeng, Kaiqiang Zheng, Xinhua He, Minqiang Xie, Xiaoyi Fu

**Affiliations:** 1 School of Materials Science and Engineering, South China University of Technology, Guangzhou 510641, China; zhaoyujing_zibo@126.com (J.Z.); zm1992123@sina.com (M.Z.); zkq0908@126.com (K.Z.); imxhhe@scut.edu.cn (X.H.); 2 Department of Otorhinolaryngology Head & Neck Surgery, Zhujiang Hospital of Southern Medical University, Guangzhou 510282, China; min_qiang_x@hotmail.com

**Keywords:** P(NIPAM-AA), Fe_3_O_4_/SiO_2_ hollow microspheres, holes, drug release

## Abstract

Thermoresponsive P(NIPAM-AA)/Fe_3_O_4_/SiO_2_ microspheres with surface holes serving as carriers were prepared using p-Fe_3_O_4_/SiO_2_ microspheres with a thermoresponsive copolymer. The p-Fe_3_O_4_/SiO_2_ microspheres was obtained using a modified Pickering method and chemical etching. The surface pore size of p-Fe_3_O_4_/SiO_2_ microspheres was in the range of 18.3 nm~37.2 nm and the cavity size was approximately 60 nm, which are suitable for loading and transporting biological macromolecules. P(NIPAM-AA) was synthesized inside and outside of the p-Fe_3_O_4_/SiO_2_ microspheres via atom transfer radical polymerization of NIPAM, MBA and AA. The volume phase transition temperature (VPTT) of the specifically designed P(NIPAM-AA)/Fe_3_O_4_/SiO_2_ microspheres was 42.5 °C. The saturation magnetization of P(NIPAM-AA)/Fe_3_O_4_/SiO_2_ microspheres was 72.7 emu/g. The P(NIPAM-AA)/Fe_3_O_4_/SiO_2_ microspheres were used as carriers to study the loading and release behavior of BSA. This microsphere system shows potential for the loading of proteins as a drug delivery platform.

## 1. Introduction

Targeted drug delivery systems are a hot topic in the fields of biology and medicine [1,2]. In previous studies, the microspheres used as carriers usually have had a pore size of 2–3 nm, which restricts the selection of drug molecules that can be loaded and their biomedical applications [3]. However, the microspheres are easily ruptured and destroyed by constructing large, dense holes in small particles with diameters of less than 200 nm. In this context, the design of small microspheres with macroporous holes that are structurally stable enough to carry large molecular drugs is an attractive and challenging subject. Two important and rapidly developing fields that will take advantage of this are peptide and gene therapy. 

In particular, among the potential delivery platform materials, such as mesoporous materials [4,5,6,7], hollow microspheres [8,9,10,11] and nanotubes [12,13] etc., Fe_3_O_4_/SiO_2_ hollow magnetic microspheres [14,15] have been widely used as carriers for the storage and delivery of cargo species due to their distinctive properties, such as facile functionalization, good biocompatibility, physical and chemical stability. Fe_3_O_4_/SiO_2_ hollow magnetic microspheres, which are often used as a carrier, usually have a pore structure, such as with big holes [16] or mesoporous [8,17], because of their higher loading capacity. Using magnetic hollow microspheres as carriers, cargo loading and release can be accomplished by constructing the appropriate pore channels. Fe_3_O_4_/SiO_2_ hollow microspheres with holes that have anisotropic properties can be called Janus particles. The methods of synthesizing Janus particles broadly employ shielding a part of particles by embedding a monolayer of particles on a substrate or on a Pickering emulsion [18] and then chemically modifying these particles in an aqueous phase. In this present, we prepared the p-Fe_3_O_4_/SiO_2_ microspheres with a modified Pickering emulsion. Additionally, the chemical composition of Fe_3_O_4_/SiO_2_ microspheres assures the efficient attachment of organic surface functionalities such as stimuli-responsive polymer to improve the loading capacity of cargos. The combination of a temperature sensitive polymer and Fe_3_O_4_/SiO_2_ particles can be used as gated carriers that release cargo in response to the ambient temperature, which has potential application in magnetic hyperthermia (42 °C–49 °C) [19,20]. P(NIPAM-AA) [21,22,23] has been widely studied as a temperature sensitive polymer with good biocompatibility and stimulation response. In aqueous solutions, the lower critical solution temperature (LCST) of P(NIPAM-AA) can be controlled (32 °C–49.5 °C) [24]. In an aqueous, the polymer undergoes hydrophilic–hydrophobic transition at LCST. Similarly, the cross-linked microspheres obtained by this polymer swell in water under a critical temperature and collapse above it, and this temperature is the volume phase transition temperature (VPTT) [25,26]. In previous studies, P(NIPAM-AA) was usually grafted onto the external surface of inorganic microspheres [22] and the adsorption and desorption of cargos depended on the state of the P(NIPAM-AA) network, while coating the P(NIPAM-AA) into the cavity of the hollow microspheres can largely improve the loading capacity and reduce the release rate. However, reports on the design and preparation of hollow microsphere with PNIPAM or P(NIPAM-AA) inside the cavity are limited.

In this work, we combined the P(NIPAM-AA) with the hollow p-Fe_3_O_4_/SiO_2_ microspheres to design a stimuli-responsive drug delivery system. Despite the great potential of this strategy, the use of this composite microsphere remains largely unexplored. We developed a carrier based on bowl-like hollow p-Fe_3_O_4_/SiO_2_ microspheres with P(NIPAM-AA) serving as a gatekeeper that allows the encapsulation and transportation of drugs with little premature release to specific site tissue and organs where the drug can be released upon an external alternating magnetic field. In this paper, the surface hole size of p-Fe_3_O_4_/SiO_2_ microspheres was in the range of 18.3~37.2 nm, which allows encapsulation of big molecules of any type. The VPTT of the P(NIPAM-AA)/Fe_3_O_4_/SiO_2_ microsphere was 42.5 °C, which meets the requirements for magnetic hyperthermia. The protein BSA was used as a model drug to study the loading and release properties of the P(NIPAM-AA)/Fe_3_O_4_/SiO_2_ microspheres.

## 2. Materials and Methods

### 2.1. Materials

*N*-isopropyl-acrylamide (NIPAM, 98%) and ammonium persulfate (APS) were purchased from Aladdin Chemistry Co., Ltd. (Shanghai, China). *N*,*N*′-methylene-bis(2-propenamide) (MBA) was purchased from Tianjin Damao Chemical Co., Ltd. (Tianjin, China). Bovine serum albumin (BSA) was obtained from Sigma-Aldrich. Sodium acetate (CH_3_COONa) and trichloromethane (CH_3_Cl) were purchased from Guangzhou Chemistry Co., Ltd. (Guangzhou, China). Ethylene glycol (EG), trisodium citrate dehydrate (Na_3_Cit) and acrylic acid (AA) were purchased from Shanghai Runjie Chemical Reagent Co., Ltd. (Shanghai, China). Paraffin Wax (melting point 60–62 °C) was purchased from Shanghai Yonghua Paraffin Co., Ltd. (Shanghai, China). Ferric trichloride (FeCl_3_·6H_2_O) was purchased from Big Alum Chemical Reagent Factory. Tetraethoxysilane (TEOS) was obtained from China Wulian Chemical Co., Ltd. Sodium hydroxide (NaOH) and oxalic acid (C_2_H_2_O_4_·2H_2_O) were purchased from Tianjin Qilun Chemical Technology Ltd. (Tianjin, China). Absolute ethyl alcohol (C_2_H_5_OH) was obtained from the Tianjin Fuyu Chemical Co., Ltd. (Tianjin, China). Ammonium hydroxide (NH_3_·H_2_O, 25%) was purchased from China Sun Specialty Products Co., Ltd. (Jiangsu, China).

### 2.2. Preparation of the p-Fe_3_O_4_/SiO_2_ Microspheres

*Synthesis of Fe_3_O_4_ microspheres*. The Fe_3_O_4_ hollow microsphere were prepared via a solvothermal method [27]. FeCl_3_·6H_2_O (2.16 g) and 0.08 g Na_3_Cit were dissolved in 64 mL of EG. Then, 5.76 g CH_3_COONa was added to the mixture until the mixture was completely dissolved. The solution was transferred into a 100 mL Teflon-lined autoclave, which was sealed and maintained at 200 °C for 15 h. The precipitation was washed with deionized water and ethanol several times, and dried in a vacuum oven at 60 °C for 12 h.

*Synthesis of Fe_3_O_4_/SiO_2_ microspheres*. A layer of silica was coated on the Fe_3_O_4_ microspheres using a modified StÖber method [28] to form the Fe_3_O_4_/SiO_2_ microspheres. In a typical synthesis, the Fe_3_O_4_ microspheres (100 mg) were suspended in a mixture of 20 mL ethanol, 2 mL deionized water and 0.2 mL aqueous ammonia solution. After ultrasonication for 15 min, the solution was stirred for 30 min. Then, 30 μL of TEOS was added to the mixture and allowed to react for 10 h. The precipitant was washed with ethanol several times and dried.

*Synthesis of p-Fe_3_O_4_/SiO_2_ microspheres*. The Fe_3_O_4_/SiO_2_ microspheres (50 mg) were suspended in 20 mL of deionized water and heated to 80 °C, then 300 mg of wax were added. After the wax completely melted, the solution was stirred vigorous for 15 min, the composite particles of wax/Fe_3_O_4_/SiO_2_ were produced and collected with a magnet. The obtained wax/Fe_3_O_4_/SiO_2_ composite particles were suspended in a 40 mL NaOH solution (1 mol/L) for 24 h to partly etch the SiO_2_. To release the Fe_3_O_4_/SiO_2_ microspheres that were partly coated with a SiO_2_ shell from the wax, the produced composite particles were suspended in CHCl_3_ (10 mL) for 12 h at room temperature and washed for three times to remove wax completely. The obtained microspheres were washed with ethanol and deionized water several times. To produce the Fe_3_O_4_/SiO_2_ microspheres with surface holes, the obtained microspheres were suspended in 20 mL of an oxalic acid solution (0.025 mol/L) for 15 min, 30 min, 45 min and 60 min. Then the p-Fe_3_O_4_/SiO_2_ microspheres were obtained after being washed with ethanol and dried in a vacuum oven at 60 °C for 12 h.

### 2.3. Synthesis of the P(NIPAM-AA)/Fe_3_O_4_/SiO_2_ Microspheres

p-Fe_3_O_4_/SiO_2_ (100 mg) microspheres with different pore sizes were suspended in 50 mL of deionized water and ultrasonicated for 30 min. Then, AA was added into the suspended solution, and the mixture was heated to 70 °C under a N_2_ atmosphere [29]. After reacting for 4 h, NIPAM, MBA and SDS were added sequentially and stirred for 30 min, then the APS solution was injected slowly into the mixture and stirred for 20 h to produce the P(NIPAM-AA)/Fe_3_O_4_/SiO_2_ microspheres. The produced microspheres were washed with deionized water and ethanol sequentially, and dried in a vacuum oven at 60 °C for 12 h. The addition of AA, NIPAM, MBA, SDS and APS is shown in Table 1.

### 2.4. Loading and Release 

The loading and release of BSA from p-Fe_3_O_4_/SiO_2_ and P(NIPAM-AA)/Fe_3_O_4_/SiO_2_ microspheres was examined. The P(NIPAM-AA)/Fe_3_O_4_/SiO_2_ microspheres (50 mg) were suspended in a 10 mL of BSA/H_2_O solution (1.5 g/L). After stirring for 24 h at 30 °C, the BSA-loaded microspheres were centrifuged, and the supernatant was used to calculate the BSA loading efficiency. The absorbance of the samples was measured using a UV-Vis spectrophotometer at 280 nm. The loading efficiency is quantified by Equation (1):

(1) $$\mathrm{Loading}\;\mathrm{efficiency}\;\left(\mathrm{mg}\cdot g^{-1}\right)=\frac{\mathrm{Quantity}\;\mathrm{of}\;\mathrm{BSA}\;\mathrm{on}\;P\left(\mathrm{NIPAM}-\mathrm{AA}\right)/{\mathrm{Fe}}_{3}O_{4}/{\mathrm{SiO}}_{2}\;\left(\mathrm{mg}\right)}{\mathrm{Quantity}\;\mathrm{of}\;P\left(\mathrm{NIPAM}-\mathrm{AA}\right)/{\mathrm{Fe}}_{3}O_{4}/{\mathrm{SiO}}_{2}\;\left(g\right)}$$

The BSA-loaded microspheres (50 mg) were suspended in 10 mL of a phosphate-buffered saline (PBS) solution at pH = 7.4 with continuous shaking. At specific time intervals, a 3-mL solution was sampled and replaced with an equal volume of the fresh releasing medium. The BSA concentration was measured using a UV-vis spectrometer at 280 nm, and the cumulative release rate of the BSA was calculated from the standard curve of the BSA. The BSA release experiments were performed at 37 °C and 47 °C, respectively. The cumulative release rate is quantified by Equation (2):

(2) $$\mathrm{Cumulative}\;\mathrm{release}\;\mathrm{rate}\;\left(\%\right)=\frac{\mathrm{Drug}\;\mathrm{release}\;\mathrm{capacity}\;\mathrm{of}\;P\left(\mathrm{NIPAM}-\mathrm{AA}\right)/Fe_{3}O_{4}/\mathrm{Si}O_{2}}{\mathrm{Drug}\;\mathrm{laoding}\;\mathrm{capacity}\;\mathrm{of}\;P\left(\mathrm{NIPAM}-\mathrm{AA}\right)/Fe_{3}O_{4}/\mathrm{Si}O_{2}}*100\%$$

### 2.5. Characterizations

The crystal microstructures of the products were identified by X-ray diffraction (XRD; X’Pert Pro, Philips, Amsterdam, The Netherlands) using Cu Kα radiation with a wavelength of 0.154 nm. The morphologies of the products were characterized using field emission scanning electron microscopy (Nova NanoSEM 430 m, FEI, Czech Republic, The Netherlands). TEM observation was performed on a transmission electron microscope (CM300, Philips, Amsterdam, The Netherlands). We use the Nano Measurer software to measure the size, porosity and surface holes of the Fe_3_O_4_, Fe_3_O_4_/SiO_2_, p-Fe_3_O_4_/SiO_2_ microspheres. More than 200 microspheres were counted and sized to determine the average particle size, porosity, surface holes and cavity microspheres. The Fourier transform infrared spectroscopy (FT-IR) spectra of the microspheres were measured using a spectrometer (Vector 33-MIR, Brukev Optic, Ettlingen, Germany) ranging from 400 to 4000 cm^−1^ with a KBr pellet technique. The weight loss behaviors for the microspheres were examined using thermogravimetry (TGAR5000IR, Quantachrome, FL, USA). These samples were heated from room temperature to 600 °C at 10 °C/min in N_2_. Differential scanning calorimetry (DSC) was conducted using the DSC (DSC 214 polyma, Netzsch, Bavarian Asia, Germany). The samples were dispersed in deionized water and heated at a rate of 5 °C per minute and scanned in a range of 20 °C to 80 °C in N_2_. The magnetic property of the sample was investigated on a PPMS-9 (Qyantum Design Inc., San Diego, CA, USA) with a vibrating sample magnetometer (VSM). UV-visible spectra were recorded using a lambda 35 Spectrometer. The BET analysis was performed on an Autosorb IQ (Quantachrome, FL, USA). Zeta potential was measured by using SZ-100 (HORIBA, Kyoto, Japan). Dynamic Light Scattering (DLS) measurements were carried by using nano particle analyzer SZ-100 (HORBIA, Kyoto, Japan). Suspensions of nanoparticles (0.1% *w*/*v*) were prepared in deionized water and sonicated for 20 min before the analysis. The measurements of hydrodynamic diameter (HD) were performed in triplicate for each sample and the mean value is reported. 

## 3. Results and Discussion

**Figure 1:**
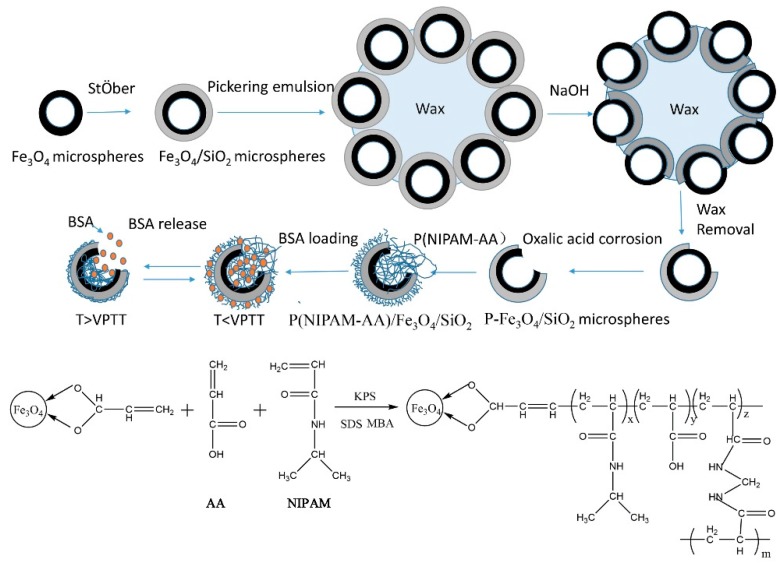
Preparation of the P(NIPAM-AA)/Fe_3_O_4_/SiO_2_ microspheres with surface holes and the BSA loading and release schematic.

Figure 1 shows the preparation of the P(NIPAM-AA)/Fe_3_O_4_/SiO_2_ microspheres and their BSA-loading and release profiles. First, the Fe_3_O_4_/SiO_2_ hollow microspheres were prepared using a solvothermal method and a modified StÖber method. Then, Fe_3_O_4_/SiO_2_ microspheres were partly embedded in wax to form wax/Fe_3_O_4_/SiO_2_ particles via the Pickering emulsion method [5]. Subsequently, the unprotected SiO_2_ shell was etched by an aqueous solution of NaOH. Then Fe_3_O_4_/SiO_2_ microspheres partly coated with SiO_2_ shell were obtained after etching wax. When the microspheres were immersed in oxalic acid solution, the unprotected Fe_3_O_4_ was etched quickly, and holes were generated. Finally, microspheres with different pore sizes (represented by p-Fe_3_O_4_/SiO_2_) can be obtained by controlling the oxalic acid treatment time. Before addition of initiator (KPS), the –COOH of AA can reacted only with –OH of Fe_3_O_4_, which can promote the synthesis of P(NIPAM-AA) into the cavity of p-Fe_3_O_4_/SiO_2_ microspheres, subsequently. Then the P(NIPAM-AA)/Fe_3_O_4_/SiO_2_ microspheres were synthesized via atom transfer radical polymerization of NIPAM, MBA, AA. The obtained P(NIPAM-AA)/Fe_3_O_4_/SiO_2_ composite microspheres can be used as drug carriers to load biological macromolecules (BSA). BSA was loaded below the VPTT and released when the temperature was higher than the VPTT.

### 3.1. The Preparation and Morphology Characterization of the p-Fe_3_O_4_/SiO_2_ Microspheres

**Figure 2:**
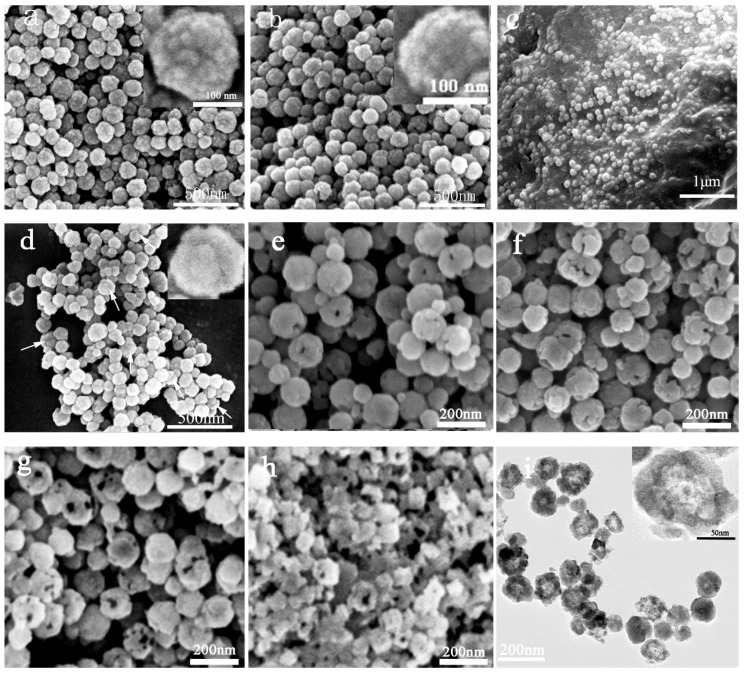
The SEM and TEM images of the microspheres obtained during the preparation process. (**a**) Fe_3_O_4_; (**b**) Fe_3_O_4_/SiO_2_; (**c**) wax/Fe_3_O_4_/SiO_2_ (**d**) Fe_3_O_4_ microspheres partly coated with a SiO_2_ shell; (**e**–**h**) p-Fe_3_O_4_/SiO_2_ after oxalic acid corrosion for different times ((**e**) 15 min; (**f**) 30 min; (**g**) 45 min; (**h**) 60 min) (**i**) TEM image of the Fe_3_O_4_/SiO_2_ microspheres.

Figure 2 shows the SEM and TEM images of the microspheres obtained during the preparation process. Figure 2a shows that the Fe_3_O_4_ microspheres are spherical and well dispersed, and the surface of the microspheres is coarse and has small granular protrusions, which indicate that the Fe_3_O_4_ microspheres are composed of Fe_3_O_4_ nanoparticles [27]. The average particle size of the Fe_3_O_4_ microspheres is 114 ± 5 nm (Size distribution histogram of Fe_3_O_4_ and the Fe_3_O_4_/SiO_2_ microspheres shown in Supplementary Information Figure S1). After coating the SiO_2_ layer, the mean diameter of the Fe_3_O_4_/SiO_2_ microspheres is 125 ± 7 nm and the thickness of the silica coating was estimated to be 6 nm. After the Pickering emulsification, the wax/Fe_3_O_4_/SiO_2_ particles with diameter in arrange of 20 μm–40 μm (Supplementary Information Figure S2) were formed by the Fe_3_O_4_/SiO_2_ microspheres partially embedded in paraffin, and Fe_3_O_4_/SiO_2_ microspheres were homogeneously distributed on the paraffin surface (Figure 2c). Figure 2d shows that the original uniform spherical shape becomes mushroom-like and exhibits a Janus structure: the Fe_3_O_4_ microspheres partly coated with the SiO_2_ shell. The formation of the Janus microspheres is attributed to the etching of the SiO_2_ shell layer by NaOH. The p-Fe_3_O_4_/SiO_2_ microspheres were obtained after etching for 15 min (Figure 2e), 30 min (Figure 2f) and 45 min (Figure 2g), and the porosity was 47%, 59%, and 76%, respectively; the corresponding average surface holes diameter was approximately 20.3 ± 5 nm, 28.3 ± 8 nm and 37.2 ± 7 nm. The porosity, the pore size and the number of surface holes increase with increasing etching time. However, most of the microspheres were broken when the etching time was 60 min (Figure 2h). Figure 2i shows that most of the microspheres have a hollow structure. The size of the Fe_3_O_4_/SiO_2_ microspheres is approximately 125 ± 7 nm, and the average size of the cavity is approximately 60 nm.

### 3.2. Porous Structure Characterization of the p-Fe_3_O_4_/SiO_2_ Microspheres

**Figure 3:**
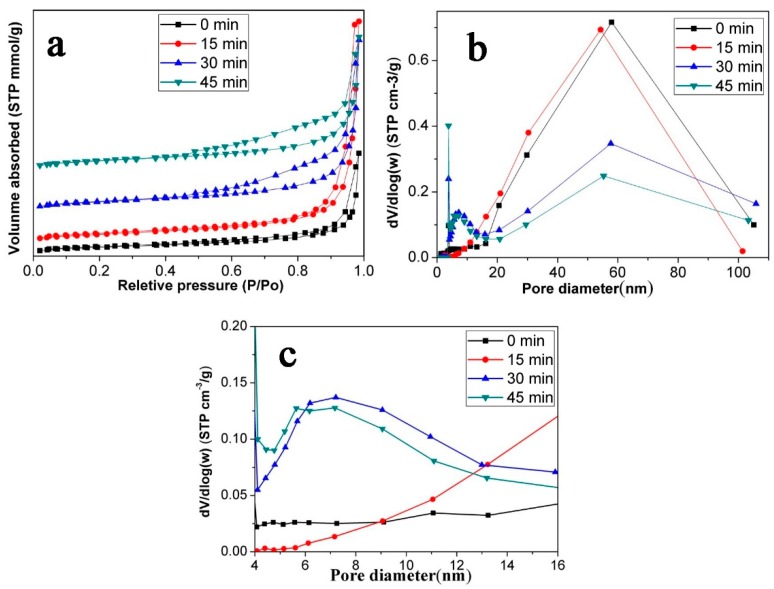
The adsorption/desorption isotherms (**a**) and the corresponding BJH pore size distribution for the Fe_3_O_4_/SiO_2_ and p-Fe_3_O_4_/SiO_2_ microspheres (**b**,**c**).

To study the pore structure of the p-Fe_3_O_4_/SiO_2_ microspheres, N_2_ adsorption/desorption experiments were performed. Figure 3a shows the N_2_ adsorption/desorption isotherms for the Fe_3_O_4_/SiO_2_ microspheres and p-Fe_3_O_4_/SiO_2_ microspheres etched with oxalic acid with different times. All the isotherms are type IV adsorption isotherms, as defined by IUPAC [30]. The BET surface area of the Fe_3_O_4_/SiO_2_ microspheres without oxalic acid etching was 32.9 m^2^/g. After etching for 15 min, 30 min and 45 min, the BET surface areas of the p-Fe_3_O_4_/SiO_2_ microspheres were 38.3 m^2^/g, 42.2 m^2^/g and 39.6 m^2^/g, respectively, which shows an increase compared to the un-etched microspheres. However, the surface areas of the microspheres prepared at various etching times are similar. We suspect this is because the surface holes are enlarged, which decreases the specific surface area as the oxalic acid continues to etch. However, the formation of new pores increase the surface area. For these two aspects, the surface area of the microspheres see little change with increased etching time.

The pore size distribution of microspheres mainly ranged from 10 nm–100 nm (Figure 3b). The pore size that appeared near 60 nm could be associated with microsphere cavities, which agrees with the TEM images. In addition, the p-Fe_3_O_4_/SiO_2_ microsphere at 30 min and 45 min had a pore size distribution from 4 nm to 10 nm (Figure 3c). It is obvious to find that the surface areas of microspheres at 30 min and 45 min are similar, but the porosity at 45 min is higher. The higher the porosity, the higher the loading capacity, which is consistent with our following experimental results. Thus, we focus on the study of p-Fe_3_O_4_/SiO_2_ microspheres which were etched for 45 min by oxalic acid grafting with P(NIPAM-AA).

### 3.3. Morphology Characterization of the P(NIPAM-AA)/Fe_3_O_4_/SiO_2_ Microspheres

**Figure 4:**
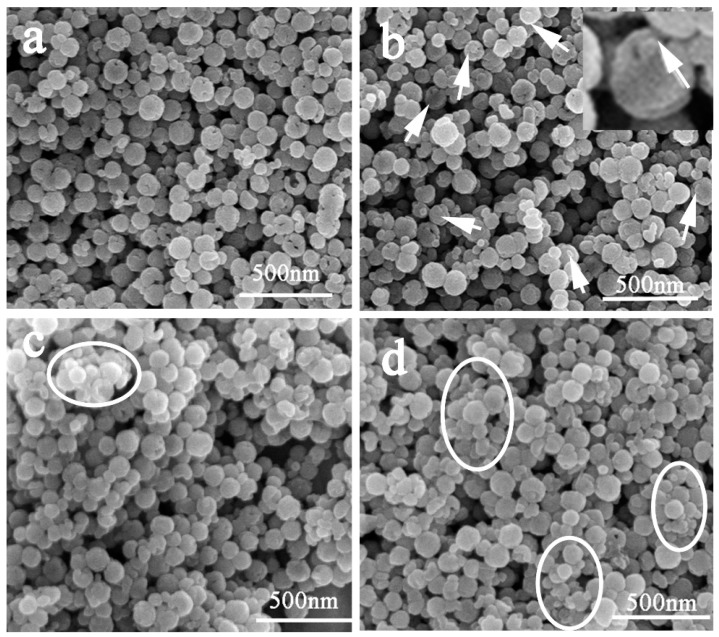
SEM images and HD distribution curves of the P(NIPAM-AA)/Fe_3_O_4_/SiO_2_ microspheres prepared with various amounts of NIPAM, MBA, AA. (**a**) Sample A1; (**b**) sample A2; (**c**) sample A3 and (**d**) sample A4.

The SEM images of the P(NIPAM-AA)/Fe_3_O_4_/SiO_2_ microspheres obtained (under different amount of monomer addition shown in Table 1) are shown in Figure 4. The P(NIPAM-AA)/Fe_3_O_4_/SiO_2_ microspheres were produced via atom transfer radical polymerization of NIPAM, MBA and AA, after the esterification reaction between AA and p-Fe_3_O_4_/SiO_2_ (etching for 45 min) microspheres. It is interesting that some small protrusions appeared in the holes (marked with arrows in Figure 4b). We suspect that this was because P(NIPAM-AA) was grafted into the holes of p-Fe_3_O_4_/SiO_2_ microspheres. The size of microspheres increased with increasing amounts of monomers, the HD of p-Fe_3_O_4_/SiO_2_ microspheres was 102 ± 9 nm, and after grafting P(NIPAM-AA), the corresponding values were 120 ± 17 nm (sample A1) and 167 ± 21 nm (sample A2) (Supplementary Information Figure S3). The HD distribution curves also show the good dispersibility of samples A1 and A2. From Figure 4b–d, with the increasing amount of monomers, the holes gradually disappeared, and big aggregated particles also appeared (marked with circles in Figure 4c,d), which can be proved by HD results (Supplementary Information Figure S3) [31]. 

### 3.4. Characterization of the P(NIPAM-AA)/Fe_3_O_4_/SiO_2_ Microspheres

**Figure 5:**
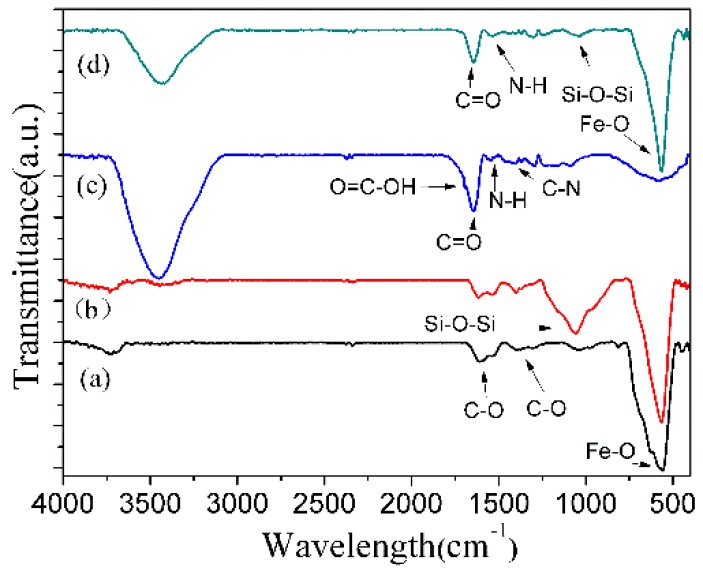
Infrared spectra of (**a**) Fe_3_O_4_; (**b**) Fe_3_O_4_/SiO_2_; (**c**) P(NIPAM-AA) and (**d**) P(NIPAM-AA)/Fe_3_O_4_/SiO_2_ microspheres.

Figure 5 presents the FTIR spectra of Fe_3_O_4_, p-Fe_3_O_4_/SiO_2_, P(NIPAM-AA) and P(NIPAM-AA)/Fe_3_O_4_/SiO_2_ microspheres. The FTIR spectrum of Fe_3_O_4_ microspheres showed the peak at 567 cm^−1^ is attributed to the stretching Fe–O of Fe_3_O_4_ and at 1622 cm^−1^ and 1389 cm^−1^ is attributed to the anti-symmetrical and symmetric vibration of COO^−^ which indicate the existence of carboxylate groups on the Fe_3_O_4_ microspheres. The characteristic band at 1097 cm^−1^ is attributed to the Si–O–Si vibration of the FTIR spectrum of Fe_3_O_4_/SiO_2_ microspheres. The FTIR spectrum of P(NIPAM-AA)/Fe_3_O_4_/SiO_2_ microspheres showed the characteristic peaks at 1537 cm^−1^ (stretching peak of N–H), 1650 cm^−1^ (stretching peak of C=O) and 1394 cm^−1^ (C–N stretching peak), which confirmed that P(NIPAM-AA) was successfully grafted to the p-Fe_3_O_4_/SiO_2_ microspheres.

**Figure 6:**
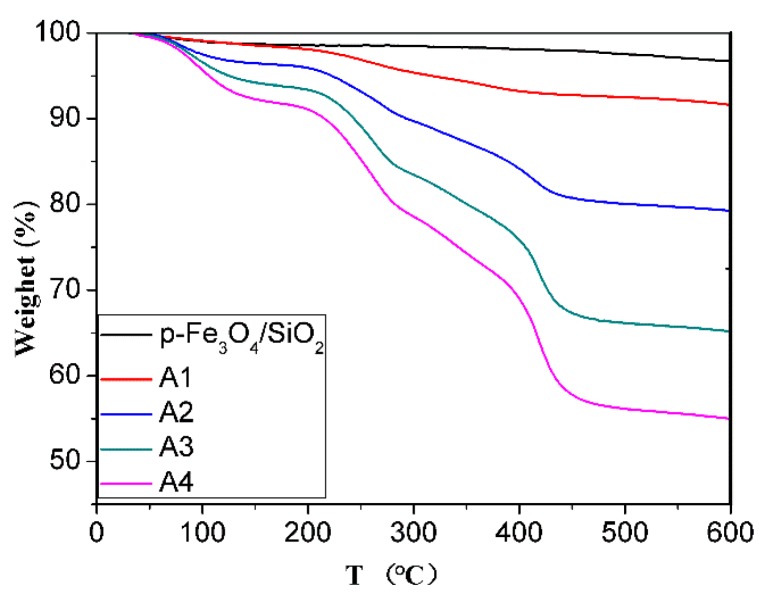
TGA curves of the p-Fe_3_O_4_/SiO_2_ microspheres and P(NIPAM-AA)/Fe_3_O_4_/SiO_2_ microspheres.

The organic compound content in the composite microspheres were determined using TGA measurements, as shown in Figure 6. In all cases, the weight loss below 200 °C can be ascribed to desorption of the physically bonded small molecule monomers and water. The weight loss in the 200 °C–450 °C temperature range can be attributed to the decomposition of the polymer, and the residual weight can be ascribed to Fe_3_O_4_ and SiO_2_. For samples A1, A2, A3 and A4, the weight loss of the polymer was 5.33%, 13.18%, 26.03% and 33.28%, respectively, and the corresponding weight ratio of inorganic/polymer of 17.76, 6.26, 2.83, 2.00, suggesting that the proportion of the polymer in the composite microspheres increased with increasing amounts of monomer.

**Figure 7:**
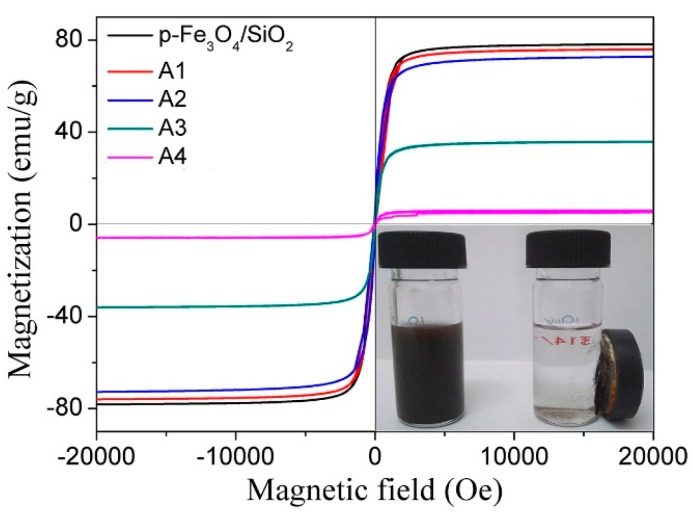
The VSM curves for the p-Fe_3_O_4_/SiO_2_ microspheres and P(NIPAM-AA)/Fe_3_O_4_/SiO_2_ composite microspheres prepared under various conditions.

The room temperature magnetization hysteresis curve of the p-Fe_3_O_4_/SiO_2_ microspheres (Figure 7) exhibits negligible hysteresis, which indicates that the microspheres are superparamagnetic. For samples A1–A3, the magnetization curves of the P(NIPAM-AA)/Fe_3_O_4_/SiO_2_ microspheres was similar to that of the bare p-Fe_3_O_4_/SiO_2_ microspheres, which indicated that the grafting polymer can preserve their superparamagnetic properties. For sample A4, the magnetization curve the P(NIPAM-AA)/Fe_3_O_4_/SiO_2_ microspheres suggest the microspheres was non-superparamagnetic and we suspected that was due to formation of Fe_2_O_3_ via the partial oxidation of Fe_3_O_4_. Fe_2_O_3_ is superparamagnetic when the particle size is smaller than 20 nm [32,33,34]. However, the diameter of Fe_3_O_4_ is about 114 nm, so the crystal structures of the p-Fe_3_O_4_/SiO_2_ microspheres were analyzed via XRD (Supplementary Information Figure S4). All the diffraction peaks agreed with the magnetic cubic structure of Fe_3_O_4_ (JCPDS No. 19-0629). The grain size of the prepared Fe_3_O_4_ microspheres calculated by the Scherrer formula at the (311) plane was 20.8 nm, which indicated that the microspheres had a secondary structure [27]. 

The saturation magnetization (M_s_) values of the p-Fe_3_O_4_/SiO_2_ microspheres and P(NIPAM-AA)/Fe_3_O_4_/SiO_2_ composite microspheres A1, A2, A3, and A4 were 78.2, 75.8, 72.7, 35 and 6 emu/g, respectively. The M_s_ values of the P(NIPAM-AA)/Fe_3_O_4_/SiO_2_ microspheres decreased rapidly with the increase of the proportion of polymer, which was consistent with the TGA results. The prepared P(NIPAM-AA)/Fe_3_O_4_/SiO_2_ microspheres could be easily separated using a magnet (inset photographs), which demonstrated their strong magnetic response.

The microspheres used as carrier should have good dispersibility. Also, the higher of the content of polymer, the higher of the loading capacity. Therefore, sample A2 was used as a carrier for the subsequent loading and release of the protein. 

**Figure 8:**
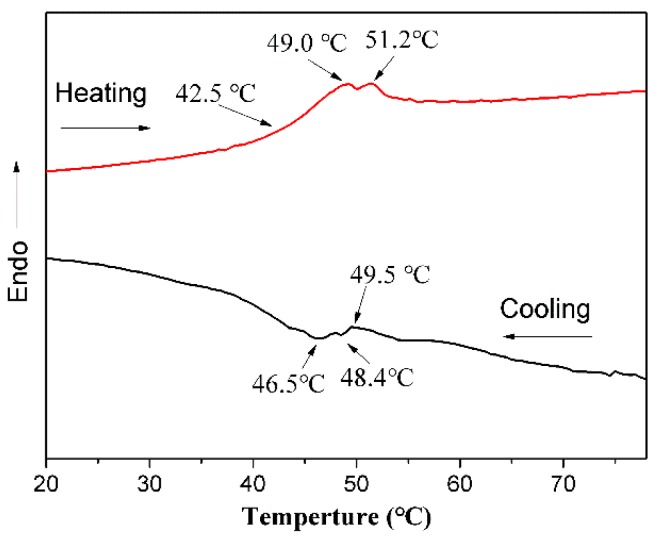
DSC curve of the P(NIPAM-AA)/Fe_3_O_4_/SiO_2_ composite microspheres (Sample A2).

A volume phase transition temperature (VPTT) is one of the most important property for the cross-linked composite particles obtained from the thermosensitive polymer [26,35,36]. Therefore, the VPTT of sample A2 microspheres was determined by using DSC measurements. The DSC curves of the P(NIPAM-AA)/Fe_3_O_4_/SiO_2_ microspheres (Figure 8) show that the peak onset temperature was observed at approximately 42.5 °C (heating curve) and 49.5 °C (cooling curve), which are the transition temperatures (VPTT) [25,26].

**Figure 9:**
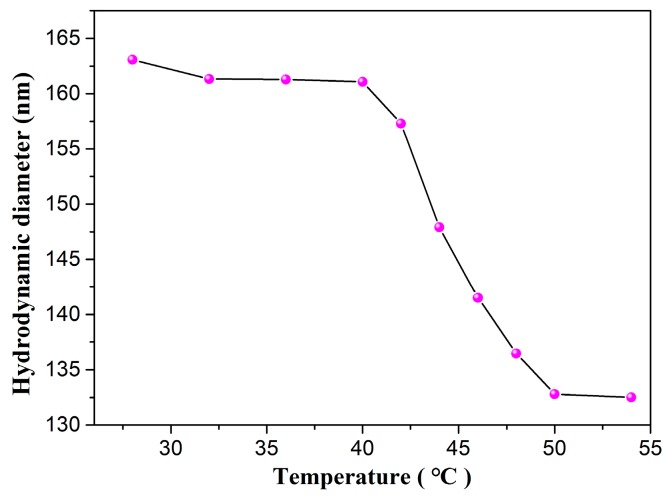
The effect of temperature on the hydrodynamic diameter of P(NIPAM-AA)/Fe_3_O_4_/SiO_2_ microspheres of sample 2.

P(NIPAM-AA) chains can also be absorbed onto the surface of the p-Fe_3_O_4_/SiO_2_ as demonstrated by the change in the hydrodynamic diameter (HD) of the hybrid P(NIPAM-AA)/Fe_3_O_4_/SiO_2_ microspheres observed by DLS measurements. Figure 9 shows the temperature dependence of the size of the hybrid microspheres. The average HD that was approximately 162 nm at 28 °C, decreasing to 135 nm at 54 °C. At 28 °C the polymer chains are present in their full extended hydrated conformation thus increasing the overall HD of particles. When the was temperature above the VPTT of the P(NIPAM-AA), the hydrated bonding between NIPAM, AA units and water molecules is disrupted, and the extended chains of polymer shrink in the globular form. Hence, the HD of the particles decreases at this temperature. These results confirm the thermoresponsive behavior of the grafted polymer and also prove the good dispersibility of nanoparticles [37,38]. The zeta potential value for the p-Fe_3_O_4_/SiO_2_ at pH 6.8 was −21.4 mV, which upon polymer grafting was −16.8 mV. This change of zeta potential can be attributed to the fact that some of the free hydroxyl groups are consumed by the polymer [39,40].

### 3.5. BSA Loading and Release 

P(NIPAM-AA)/Fe_3_O_4_/SiO_2_ microspheres and p-Fe_3_O_4_/SiO_2_ microspheres were used for the adsorption of BSA at different temperatures (Table 2). Table 2 shows that the BSA loading capacity increased with the increasing etching time of oxalic acid and the P(NIPAM-AA) grafting. The corresponding BSA loading capacity of p-Fe_3_O_4_/SiO_2_ microspheres increased by 31.0%, 71.7% and 84.8%, respectively, but the specific surface area of the microspheres increased by 16.4%, 28.6% and 20.4%, respectively, after 15 min, 30 min and 45 min of oxalic acid etching. This may be related to the formation of large holes and the mesoporous pores caused by the oxalic acid etching, and BSA (3.5 nm [41]) can only enter the interior of the microsphere through surface holes that are large enough and adsorb on the surface. The loading of BSA increased with increases in the porosity, holes and mesoporous ratio. The BSA loading capacity was further enhanced after grafting P(NIPAM-AA) because BSAs are absorbed in the P(NIPAM-AA)/Fe_3_O_4_/SiO_2_ microspheres through hydrogen bonding and van der wale forces between carboxyl-carboxyl groups. This is because the carbonyl groups of P(NIPAM-AA) can form hydrogen bonds with the amino groups and carbonyl groups of BSA (proved by FTIR in Supplementary Information Figure S5).

**Figure 10:**
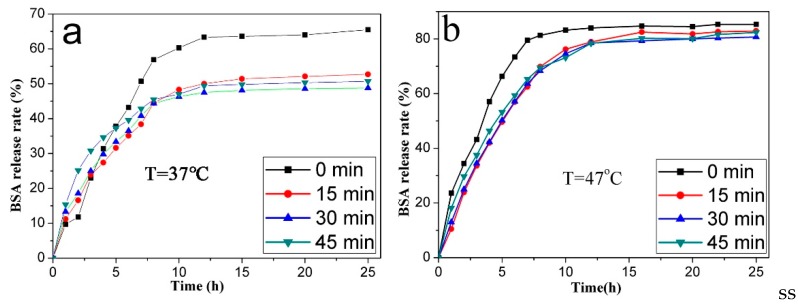
The release curves of the BSA loaded P(NIPAM-AA)/Fe_3_O_4_/SiO_2_ microspheres at 37 °C (**a**) and 47 °C (**b**).

The VPTT of P(NIPAM-AA) is 42.5 °C; thus, the release behavior of the BSA-loaded microspheres was studied at 47 °C (above the VPTT) and 37 °C (below the VPTT). Figure 10 shows that reaching the equilibrium state required lesser time and the cumulative release rate was higher when the temperature changed from 37 °C to 47 °C. Also, the cumulative release rate of BSA loaded on the P(NIPAM-AA)/Fe_3_O_4_/SiO_2_ microspheres with holes was lower than that without holes (Figure 10a).

The diffusion rate of BSA molecules adsorbed on the surface of p-Fe_3_O_4_/SiO_2_ microspheres and the polymer networks was the fastest, faster than for molucules absorbed in the mesoporous or polymer network of the microspheres. Thus, below the VPTT, the loaded BSA molecules on the surface of p-Fe_3_O_4_/SiO_2_ microspheres and polymer networks were released into the solution via diffusion (Supplementary Information Figure S6). However, above the VPTT, the BSA was released through two routes: diffusion and shrinkage extrusion. The BSA absorbed in the mesoporous or polymer network of the microspheres can be “squeezed out” when the temperature-sensitive polymer transforms from a swollen hydrophilic state into a contracted hydrophobic state. Therefore, the cumulative release rate was higher at 47 °C. 

## 4. Conclusions

Thermoresponsive P(NIPAM-AA)/Fe_3_O_4_/SiO_2_ microspheres with surface holes have been prepared by self-assembly of bowl-like hollow p-Fe_3_O_4_/SiO_2_ microspheres with P(NIPAM-AA). In this paper, p-Fe_3_O_4_/SiO_2_ microspheres had a cavity size of ~60 nm, surface holes of 18.3~37.2 nm, BET surface area about 39.6 m^2^/g and porosity up to 76%. Mesopores (4–12 nm) were also formed in the p-Fe_3_O_4_/SiO_2_ microspheres after oxalic acid etching for 30 min and 45 min. The VPTT of the prepared P(NIPAM-AA)/Fe_3_O_4_/SiO_2_ microspheres was 42.5 °C, which can meet the requirements for hyperthermia treatment, and the M_s_ was 72.7 emu/g which is very high in the current material. The BSA loading capacity can be improved after P(NIPAM-AA) was grafted inside of the cavity and outside of the p-Fe_3_O_4_/SiO_2_ microspheres. The BSA loading and release behaviors of microspheres were studied, and they suggest that the microspheres might have potential biomedical applications.

## Figures and Tables

**Table 1:** Recipe for the synthesis of the P(NIPAM-AA)/Fe_3_O_4_/SiO_2_ microspheres.

Sample 1	AA (mmol)	NIPAM (mol)	MBA (mmol)	SDS (mol)	APS (mmol)
A1	0.44	0.06	5	0.007	2.63
A2	0.88	0.12	10	0.014	3.96
A3	1.32	0.18	15	0.021	5.27
A4	1.76	0.24	20	0.028	10.52

**Table 2:** The BSA loading capacity of the microspheres at 37 °C and 47 °C.

Microspheres Corrosion Time (min)	Loading Capacity (mg/g)	
	p-Fe_3_O_4_/SiO_2_ Microspheres	P(NIPAM-AA)/Fe_3_O_4_/SiO_2_ Microspheres
0	18.4	31.6
15	24.1	41.8
30	31.6	55.2
45	34.0	74.0

## Supplementary Materials

The following are available online at www.mdpi.com/1996-1944/10/4/411/s1.
