# Supplementary material for: Preparation and Characterization of Thermoresponsive Poly(N-isopropylacrylamide-co-acrylic acid)-Grafted Hollow Fe3O4/SiO2 Microspheres with Surface Holes for BSA Release
Source: Materials (Basel). 2017 Apr 14;10(4):411. doi: 10.3390/ma10040411 (PMC5506986; doi:10.3390/ma10040411)
Supplement: Supplementary file 1 [file materials-10-00411-s001.pdf]

Supplementary Information

Preparation and Characterization of Thermoresponsive Poly(N-isopropylacrylamide-co-acrylic acid)-grafted Hollow Fe_3O_4/SiO_2 Microspheres with Surface Holes for BSA Release

Jing Zhao, Ming Zeng, Kaiqiang Zheng, Xinhua He, Minqiang Xie, Xiaoyi Fu

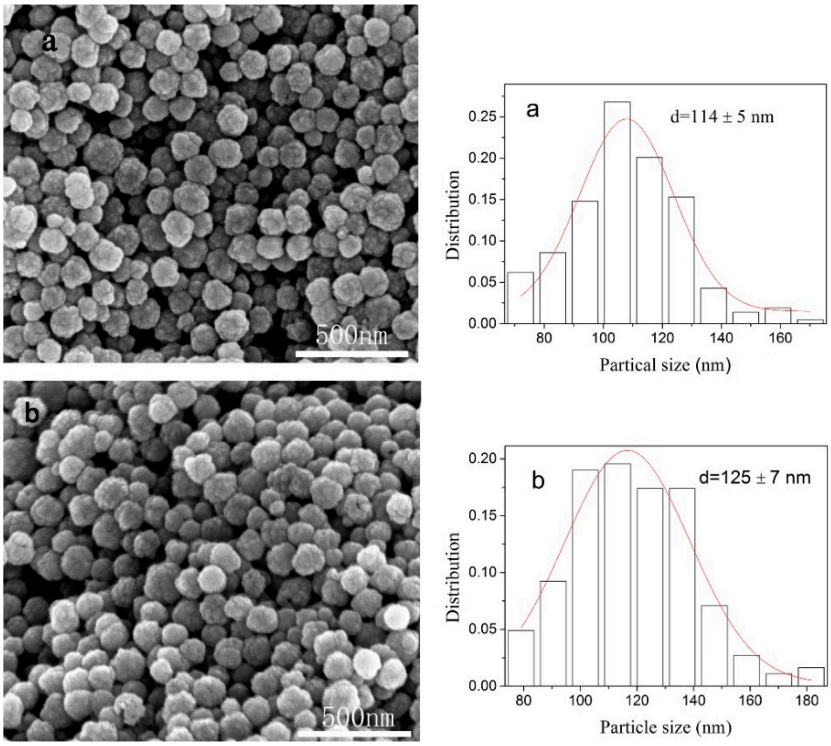

Figure S1. The SEM images and size distribution of Fe_3O_4 (a) and Fe_3O_4/SiO_2 (b) microspheres.

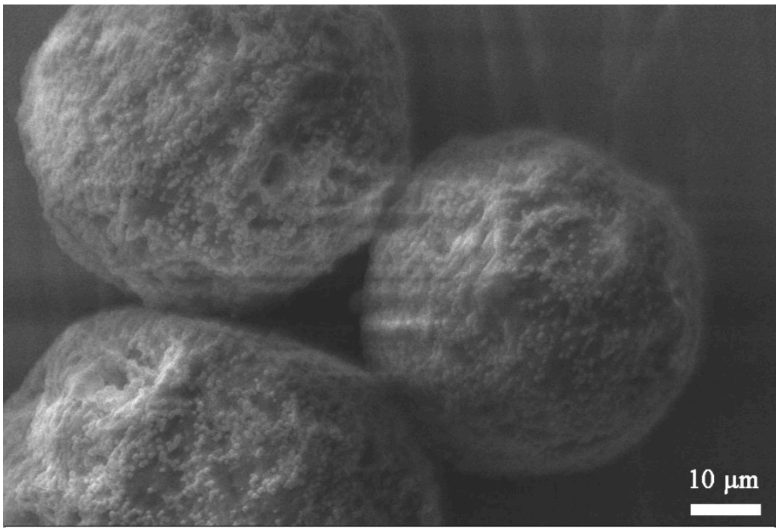

Figure S2. The SEM image of wax/Fe_3O_4/SiO_2 Pickering particles.

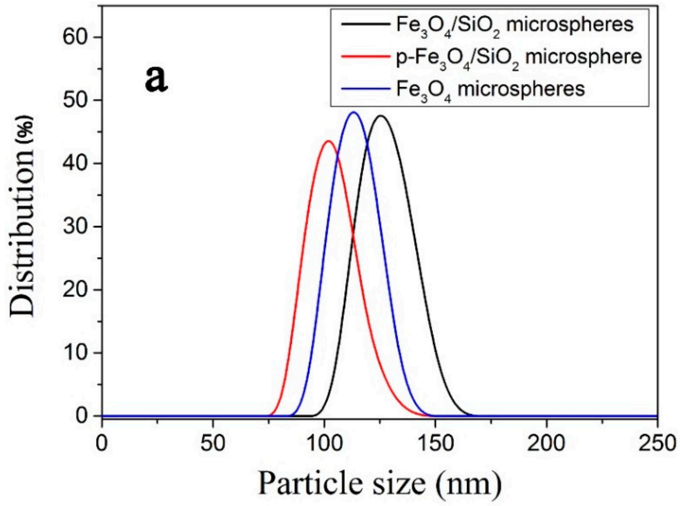

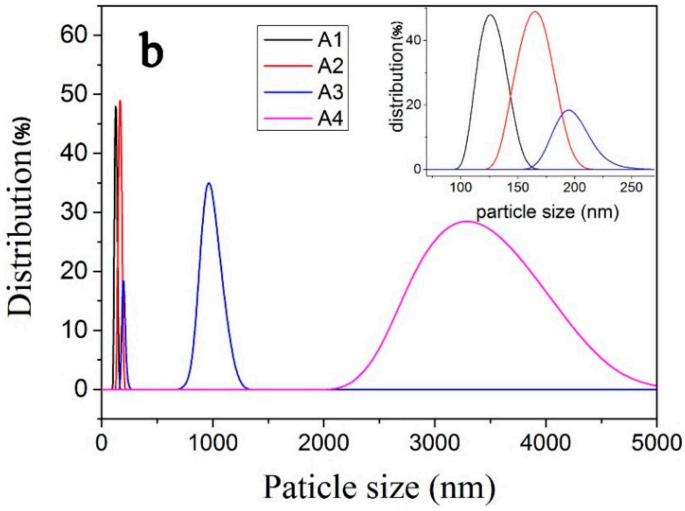

Figure S3. The hydrodynamic diameter of various microspheres at 28 ℃ (a) Fe_3O_4, Fe_3O_4/SiO_2, p-Fe_3O_4/SiO_2 microspheres, (**b**) P(NIPAM-AA)/Fe_3O_4/SiO_2 microspheres (Sample A1, A2, A3, A4).

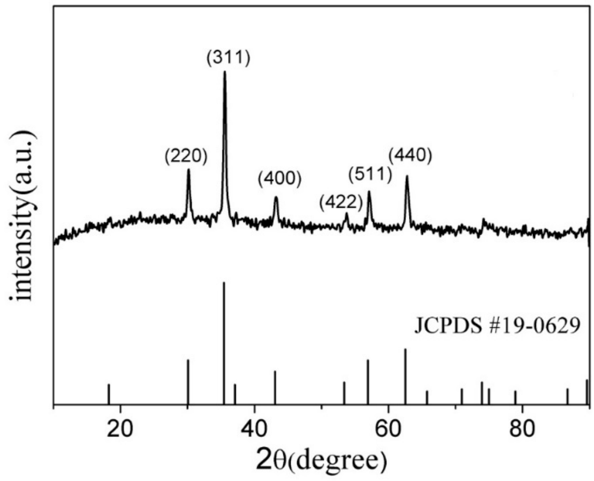

Figure S4. The XRD patterns of p-Fe_3O_4/SiO_2 microspheres.

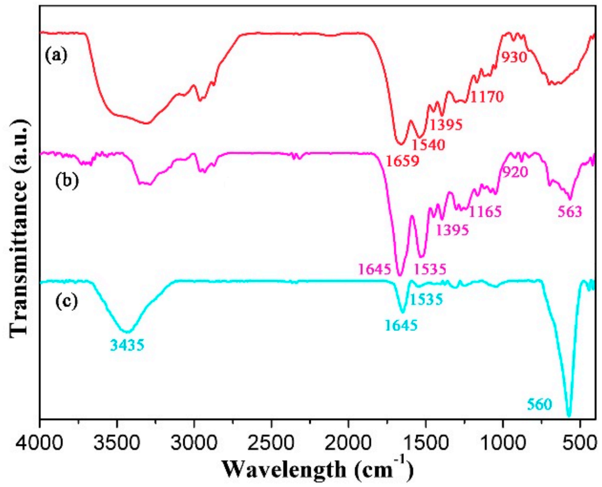

Figure S5. FTIR analysis of (**a**) BSA, (**b**) P(NIPAM-AA)/Fe_3O_4/SiO_2-BSA microspheres, (**c**) P(NIPAM-AA)/Fe_3O_4/SiO_2 microspheres.

The FTIR spectrum of BSA showed the peak with C=O (1659 cm^{-1}), N-H (1540 cm^{-1}). However, the FTIR spectrum of P(NIPAM-AA)/Fe_3O_4/SiO_2-BSA microspheres showed the peak with C=O (1645 cm^{-1}), N-H (1535 cm^{-1}). And the peak at 1170 cm^{-1}, 930 cm^{-1} are belonged to the characteristic peak of BSA which are consistent with the peak at 1165 cm^{-1}, 920 cm^{-1} of P(NIPAM-AA)/Fe_3O_4/SiO_2-BSA microspheres. These results suggested that BSA are absorbed in the P(NIPAM-AA)/Fe_3O_4/SiO_2 microspheres through hydrogen bonding and van der wale forces between carboxyl-carbonyl or amide-carboxyl groups. It is because that the carbonyl groups and amino groups of P(NIPAM-AA) can form hydrogen bond with the amino groups and carbonyl groups of BSA.

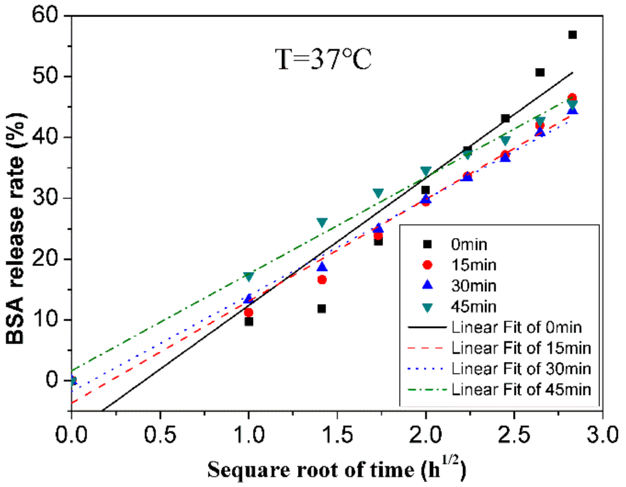

Figure S6. The fitting curve of BSA slow-release curve-Higuchi model.

Higuchi model analysis is often used to study drug release kinetics [1,2]. From Figure S6, it can be seen that the results of the release at 37 °C are analyzed by Higuchi mode. In 0h-8h segment, the correlation coefficient (R^2) of P(NIPAM-AA)/Fe_3O_4/SiO_2 microspheres obtained by oxalic acid corrosion 0min, 15min, 30min, 45min was 0.899, 0.975,0.993, 0.989, respectively; these data suggest that the diffusion control process plays a major role in the release of BSA.

References

1. Zhang W, Chai Y, Xu X, et al. Rod-shaped hydroxyapatite with mesoporous structure as drug carriers for proteins. *Appl. Surf. Sci.* **2014**, *322*, 71–77.
2. Wu J, Jiang W, Shen Y, et al. Synthesis and characterization of mesoporous magnetic nanocomposites wrapped with chitosan gatekeepers for ph-sensitive controlled release of doxorubicin. *Mater. Sci. Eng. C* **2017**, *70*, 132–140.
